# The role of diagnostic VATS in penetrating thoracic injuries

**DOI:** 10.1186/1749-7922-1-30

**Published:** 2006-10-05

**Authors:** Massimiliano Paci, Guglielmo Ferrari, Valerio Annessi, Salvatore de Franco, Guido Guasti, Giorgio Sgarbi

**Affiliations:** 1 Division of Thoracic Surgery, Azienda Ospedaliera Arcispedale Santa Maria Nuova, Viale Risorgimento, 80, 42100 Reggio Emilia, Italy; 2 Department of Anesthesiology, Azienda Ospedaliera Arcispedale Santa Maria Nuova, Viale Risorgimento, 80, 42100 Reggio Emilia, Italy

## Abstract

**Background:**

Penetrating chest injuries account for 1–13% of thoracic trauma hospital admissions and most of these are managed with a conservative approach. Nevertheless, 18–30% of cases managed only with tube thoracostomy have residual clotted blood, considered the major risk factor for the development of fibrothorax and empyema. In addition, 4–23% of chest injury patients present persistent pneumothorax and 15–59% present an injury to the diaphragm, which is missed in 30% of cases. In order to make a correct diagnosis, reduce the number of missed injuries, chronic sequelae and late mortality we propose performing surgical exploration of all patients with a penetrating injury of the pleural cavity.

**Methods:**

1270 patients who sustained thoracic trauma were admitted to our hospital between 1994 and 2004. Of these, 16 patients had penetrating injuries: thirteen were surgically explored by means of Video Assisted Thoracic Surgery (VATS), and 3 with thoracotomy due to hemodynamic instability or suspected lesion of the heart or great vessels.

**Results:**

In the 13 patients who underwent VATS, 5 injuries to the diaphragm, 3 lesions to an intercostal artery, and 1 lesion to the diaphragmatic artery were detected. In 12 of these patients a laceration of the pulmonary parenchyma was also present. A conversion to thoracotomy was necessary due to a broad laceration of the diaphragm and due to hemostasis of an intercostal artery. In all but one case, which was later converted, diagnostic imaging missed the diagnosis of laceration of the diaphragm. There was no intra- or postoperative mortality, and average hospital stay was five days.

**Conclusion:**

VATS is a safe and effective way to diagnose and manage penetrating thoracic injuries, and its extensive use leads to a reduction in the number of missed, potentially fatal lesions as well as in chronic sequelae.

## Background

Trauma associated chest injuries account for 30–40% of hospital admissions and 20–25% of trauma associated deaths. Penetrating chest injuries account for 1–13% of the total number of these injuries. Published studies report that 85% of these injuries can be managed either by means of observation or pleural drainage, while only 15–30% of cases require surgical intervention for injuries to organs that may prove fatal [1]. We believe that surgery is indicated whenever the injury is penetrating and that a complete exploration of the chest cavity should be performed whenever there is reasonable suspicion of penetration of the pleural cavity. The source of bleeding must be correctly diagnosed, as well as chest wall injuries, lung injuries, and injuries to the mediastinum and diaphragm in order to correctly manage them and also to reduce the number of missed lesions, which cause a significant number of delayed deaths. In addition, exploration of the chest cavity proves to be useful in treating retained hemothorax and persistent air leaks; their delayed treatment lead to increased morbidity, prolonged hospital stay, and chronic sequelae. Video-assisted thoracic surgery (VATS) can be used to diagnose and manage those injuries for which, until a short time ago, a thoracotomy was required. We report our experience using VATS in penetrating chest injuries over the last 10 years.

## Materials and methods

Between March 1994 and December 2004, 1270 patients were admitted to our hospital for thoracic trauma. Sixteen of these patients had a penetrating chest injury (1.2%): 13 were initially managed with VATS, and 3 with thoracotomy due to hemodynamic instability or suspected injury to the heart or great vessels. We used VATS to explore two cases of gun shot wounds, with entry and exit wounds in the thorax, 10 stab wounds and 1 sharp object wound for which there was clinical-imaging suspicion of penetration of the chest cavity.

The anatomic distribution of injuries consisted of 5-right sided wounds and 8 left-sided wounds located between the nipple line cranially, and the costal margins caudally. All patients (12 males, 1 female, median age: 32 years) underwent clinical exams, chest radiographs, FAST ultrasound (starting in 2001) and thoracic-mediastinal computed tomography (CT). Eleven patients underwent pleural drainage in the ER: 5 for pneumothorax, 4 for hemopneumothorax, and 2 for hemothorax. Patient selection criteria for VATS were: stable or normal hemodynamic condition, the ability to tolerate single lung ventilation and maintain lateral decubitus position. Unstable patients were excluded, as were those with a hemothorax over 1500 ml if hemodynamically unstable, and those with echocardiogram- or CT- suspected injury to the heart or great vessels. VATS was performed with 3-port access and 30° scope that allowed an excellent visualization of the diaphragm.

## Results

**Figure 1:**
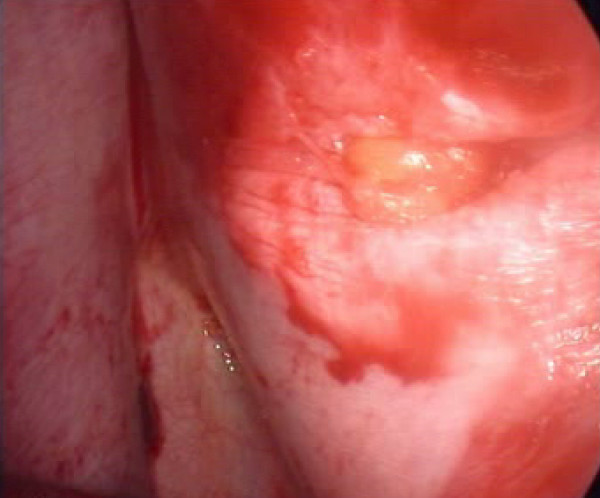
Operative photograph showing a small left diaphragmatic injury.

**Figure 2:**
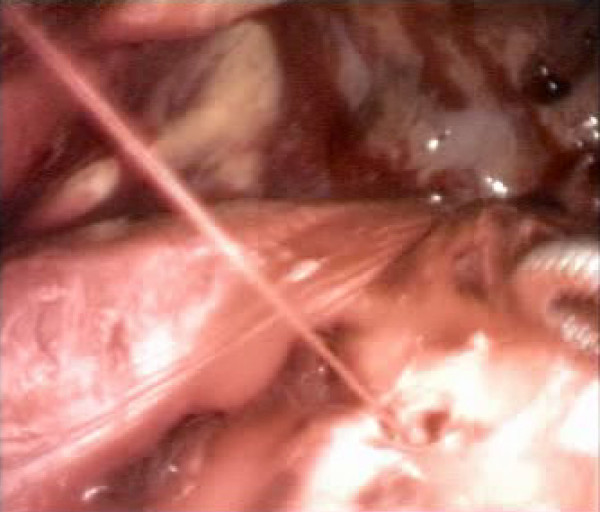
Operative photograph showing the bleeding of a right diaphragmatic artery.

**Figure 3:**
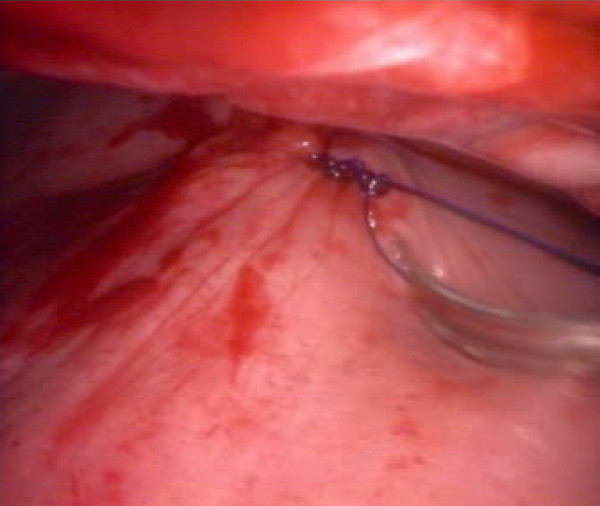
Operative photograph showing the suture of the diaphragm in VATS.

All procedures were performed within 6 hours from the moment of injury except in one case, performed after 24 hours, because the patient was initially admitted to another hospital and then sent to us because of right clotted hemothorax. We detected 5 lesions of the diaphragm (3 on the right and 2 on the left) (Fig. 1), 3 lesions of an intercostal artery, and one lesion of a diaphragmatic artery (Fig. 2). Twelve patients had a parenchymal laceration. In one patient with multiple left precordial wounds the procedure did not identify any parenchymal or vascular lesions; the hemothorax of about 800 ml was due to modest intercostal muscular bleeding. Nevertheless the procedure made it possible to exclude mediastinal vascular lesions and to assess the relationship between the wounds and the mammary vessels and pericardium. Four diaphragmatic injuries (Fig. 3), and 2 injuries to the intercostal artery were repaired using VATS. The diaphragmatic repair was performed using a non-absorbable interrupted suture. The injury to the diaphragmatic artery was repaired with non-absorbable suture by means of VATS through the lower port and all parenchymal lacerations were repaired in VATS with endoscopic staplers. Of the 13 procedures begun through VATS, 2 were converted: one in order to repair a large laceration of the right hemidiaphragm with associated hepatic injury; for the other a mini-thoracotomy had to be performed by enlarging the posterior port in order to achieve hemostasis of an intercostal artery. In 4 of the 5 diaphragmatic injuries chest CT and chest ultrasound were not able to detect the injury. There was no intra- or post-operative mortality. The mean length of hospital stay was 5 days.

## Discussion

Between 1% and 13% of trauma-related hospital admissions are due to penetrating chest injuries, the incidence of which has constantly risen over the last few years. Published studies report an exploration rate of 15–30% for the management of those injuries that immediately jeopardize the patient's life [1]. It must be remembered however that 18–30% of patients managed only with chest drainage present residual clotted blood, considered one of the main causes of the development of pulmonary atelectasis, fibrothorax, and pleural empyema (2–25%) [1-3]. In addition, in the case of penetrating injuries, the percentage of patients with persistent pneumothorax ranges from 4% to 23%, and from 15% to 59% for those with diaphragmatic injuries [1]. These latter injuries are missed in 10–30% of cases with standard diagnostic tests and are subject to a high mortality rate (36%) [2]. In our experience chest CT and ultrasound have not been able to detect injuries to the diaphragm in 4 out of 5 cases. Although exploration in only one case was negative for parenchymal and vascular injuries it did reveal the relationship between the injuries and the pericardium and mammary vessels. It must be remembered that a delayed diagnosis of a cardiac injury is associated with a high mortality rate (50%) [4]. We thus believe that surgical exploration must be performed whenever there is suspicion that a penetrating chest injury has penetrated the chest cavity in order to thoroughly examine the pleural cavity, identify the source of bleeding and possible injuries to the pulmonary parenchyma, the diaphragm, and to remove foreign bodies. In addition, the complete evacuation of blood clots and the placement of drainage under direct vision are associated with a reduction of related complications, chronic sequelae and length of hospital stay. To this end we believe that VATS can be employed as a substitute for thoracotomy whenever the patient's hemodynamic conditions permit it. Finally, VATS has an accuracy of almost 100% in diagnosing injuries to the diaphragm. Generally speaking, the rate of missed diagnosis using VATS for chest trauma is 0.8%, with a 2% rate of procedure-related complications; that for conversion is 14–31% [5,6]. Our findings are in line with those reported in published studies.

## Conclusion

It is our opinion that video-assisted thoracic surgery is safe and reliable, and is an effective alternative to thoracotomy in the management of penetrating chest injuries. In this case surgical indications should be extended so as to reduce the incidence of missed, potentially fatal injuries and to manage the source of hemothorax and persistent air leaks, whose dilation over time increases morbidity and the incidence of chronic sequelae.
